# Association of platelet-to-lymphocyte ratio with depression risk: a systematic review and meta-analysis

**DOI:** 10.3389/fpsyt.2025.1671777

**Published:** 2025-10-22

**Authors:** Ming Zhu, Wu Li

**Affiliations:** Department of Psychiatry, Shandong Daizhuang Hospital, Jining, Shandong, China

**Keywords:** platelet-to-lymphocyte ratio, depression, meta-analysis, inflammation, risk factor

## Abstract

**Background:**

The association of platelet-to-lymphocyte ratio (PLR), a simple marker of inflammation, with depressive disorders has aroused widespread attention, which, however, has not been proved by systematic evidence. Therefore, this study intends to systematically assess the association of PLR with the incidence of depressive disorders.

**Methods:**

Embase, Cochrane, PubMed, and Web of Science were searched up to April 2025 for studies investigating the association of PLR with the incidence of depressive disorders. The odds ratio (OR) or standardized mean difference (SMD) with 95% confidence interval (CI) was calculated using random-effects models. We assessed the robustness of the results and potential sources of heterogeneity by sensitivity and subgroup analyses, respectively, and evaluated publication bias by funnel plots and Egger’s test. RevMan 5.4 and Stata 18.0 were utilized for analyses.

**Results:**

Twenty-four comparative groups of 25,873 participants were included. PLR as a categorical variable was closely associated with an elevated incidence of depressive disorders (OR 1.04, 95% CI 1.00-1.08, P = 0.04), and PLR as a continuous variable was significantly higher in the depression group than in the control group (SMD 1.24, 95% CI 0.83-1.66, P<0.00001). Subgroup analyses showed a significant association of PLR with the incidence of depressive disorders in ischemic stroke and tumor patients, but this association did not reach statistical significance in children and adolescents.

**Conclusion:**

Elevated PLR is positively associated with the incidence of depressive disorders, suggesting that PLR may serve as a peripheral inflammatory indicator with potential relevance for the early identification and assessment of depressive disorders. This meta-analysis indicates that elevated PLR may be associated with depressive disorders, but substantial heterogeneity (I² = 99%) and potential publication bias warrant cautious interpretation. More large-scale prospective cohort studies across races and regions are required in the future to validate the association between PLR and the incidence of depressive disorders.

**Systematic Review Registration:**

https://www.crd.york.ac.uk/prospero/, identifier CRD420251052927.

## 1 Introduction

Depressive disorder, the most frequent and severe mental disorder, is a pervasive public health concern that has a tremendous impact on quality of life (1). Depressive disorder is usually accompanied by significant emotional changes or psychophysiologic changes, such as persistent low mood, depressed mood, loss of appetite, loss of pleasure, and slow action (2). Meanwhile, people with depression are more prone to suicide, self-harm, and even violence. Featuring high prevalence, disability, and recurrence rates, the onset of depressive disorders is associated with racial characteristics, socioeconomic status, educational level, and stress (3). Depressive disorders have a complex and highly heterogeneous etiology, and their pathogenesis remains to be clarified despite advances in the understanding of the neurobiology of depressive disorders. It has been widely recognized that the pathogenesis of depressive disorders involves multiple factors, including the interaction of environmental and genetic factors (4).

Over the past decades, research suggests that activation of systemic immunity, including abnormalities in pro-/anti-inflammatory cytokines, immune cell count, and antibody titers, is associated with depressive disorders (5). The inflammatory factor hypothesis, a research hotspot in the field of depression, holds that inflammatory factors in the peripheral blood pass through the blood-brain barrier to interfere with neurotransmitter metabolism and neuronal activity in mood-regulating brain regions, triggering depressive symptoms (6). Platelet-to-lymphocyte ratio (PLR: platelet count/lymphocyte count), an easily accessible indicator, is more sensitive than a single biochemical indicator. Decline in PLR corresponds to a decrease in platelets and a relative increase in lymphocytes, which represents the disruption of the immune-inflammatory balance. Inflammation has long been recognized as being involved in the pathophysiology of depressive disorders. Miller et al. (7) pointed out that inflammation is also associated with anxiety, irritability, and abnormal arousal (such as post-traumatic stress disorder-like symptoms), which is indicative of the body’s hypervigilant survival strategy during a “sick/injured state”. Furthermore, peripheral and central inflammatory markers (such as the brain transporter TSPO, indicating microglial activation) have significant associations with suicidal ideation. Additionally, Miller’s team found that depressive disorders with high inflammation (defined by CRP > 3 mg/L) constitute a distinct subtype, which display markedly different inflammatory marker levels from those with low inflammation (CRP ≤ 3 mg/L); they also have more pronounced core symptoms (e.g., anhedonia) and treatment characteristics (e.g., resistance to conventional antidepressants), and specific alterations in immune cell subtypes and molecular pathways, confirming the inflammation-related heterogeneity of depression (8). Wu (9) et al. believed that the effect of inflammation and immune system on the central nervous system is the basis for the association of PLR with depressive disorders, which may be involved in the neurobiological alterations in depressive disorders, and that PLR may participate in the development of depressive disorders by enhancing the release of inflammatory factors, such as interleukin-6 IL-6, and C-reactive protein. A case-control study showed that PLR is significantly higher in the depression group than in controls, and PLR is a potential biomarker of depressive disorder severity and suicide risk. A previous meta-analysis included 10 studies (2015-2021) to investigate the effect of PLR on the incidence of depressive disorders, and showed that PLR is significantly higher in depressed patients (SMD 0.24, 95% CI 0.02-0.46, P < 0.05), with high heterogeneity (I² = 88.0%) (10). Afterwards, inconsistent conclusions were reached in several new clinical studies. Therefore, we conducted this study to further assess the specific effect of PLR on the incidence of depressive disorders.

## 2 Materials and methods

### 2.1 Search strategy

This study strictly adhered to the PRISMA2020 statement (11), and the study protocol was registered with PROSPERO (CRD420251052927). Two investigators (ZM and LW) developed the search strategy, and determined independently the subject terms and keywords (“Depressive Disorder”, “Depressive Neuroses”, “Melancholia”, “Depressive Syndrome”, “Blood Platelets”, “Platelet”, “Thrombocytes”, “Lymphocytes”, “Lymphoid Cells”, “Lymphoid Cells”, and “Ratio”). Embase, PubMed, Cochrane Library, and Web of Science were searched up to April 22, 2025 (**Supplementary Table S1**).

### 2.2 Study selection

Inclusion criteria: (1) case-control (The study participants included patients with depressive disorders and healthy controls.) and cohort studies (The study participants were individuals without a history of depression.); (2) the association of PLR with incidence of depressive disorders was investigated; (3) the odds ratio (OR) or standardized mean difference (SMD) with 95% CI could be extracted directly or calculated based on available data; and (4) officially published studies in full text. Exclusion criteria: (1) reviews, conference abstracts, comments, letters, and case reports; (2) insufficient data for calculating OR or SMD with 95% CI; (3) no data; (4) overlapping or duplicate data; and (5) animal experiments.

Two investigators (ZM and LW) independently read the title and abstract of retrieved studies, and then examined the full text of potentially eligible studies. Any discrepancy was settled by consensus.

### 2.3 Data extraction

The following data were extracted independently by two investigators (ZM and LW): first author, country (study site), year of publication, study type, sample size, patient age, study duration, cutoff, OR (95% CI) for PLR, and mean and standard deviation of PLR in the depressive disorders and control groups. Any discrepancy was settled by consensus.

### 2.4 Quality assessment

The Newcastle-Ottawa Scale (NOS) (Selection, Comparability, and Outcome) was utilized for quality assessment, with a maximum score of 9 (12). Studies with 7–9 points were deemed high quality (13).

### 2.5 Statistical analysis

Data of categorical and continuous variables were synthesized by calculating OR or SMD with 95% CI. Heterogeneity was evaluated using Cochran’s Q test and Higgins *I^2^* statistic (14), and I² > 50% or P < 0.1 was deemed great heterogeneity. A random-effects model was employed. In addition, we assessed the robustness of the results and potential sources of heterogeneity by sensitivity and subgroup analyses, respectively, and evaluated publication bias by Egger’s test and funnel plots. For indicators with publication bias, the trim-and-fill method was used to assess their impact on the results. The significance level was set at P < 0.05. RevMan 5.4 and Stata 18.0 were utilized for analyses.

## 3 Results

### 3.1 Basic characteristics

**Figure 1:**
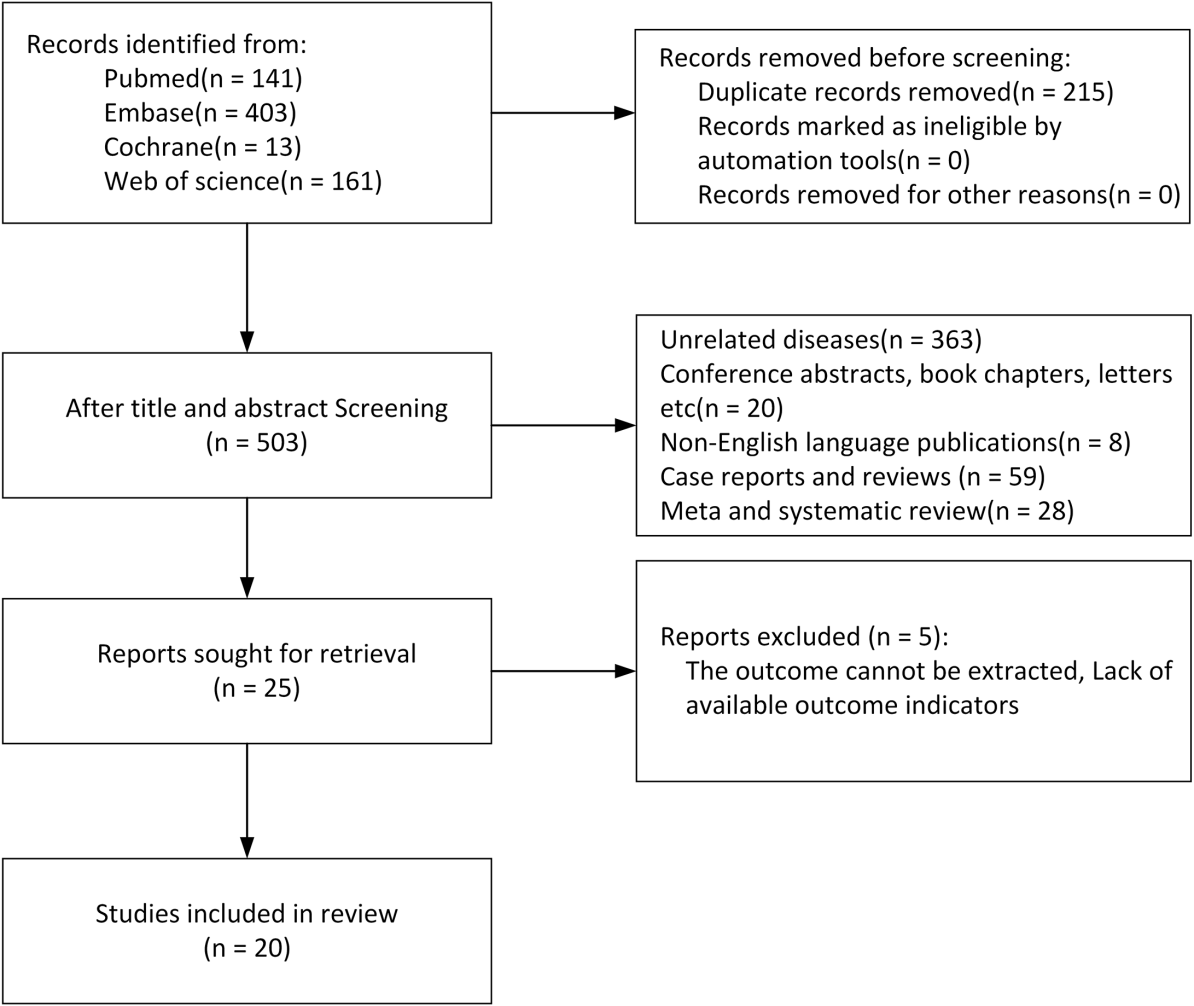
Flowchart of study screening.

Initially, 718 studies were retrieved, of which 215 duplicate publications were eliminated. After the title and abstract review, 478 studies were eliminated. Then the full text of the remaining 25 studies was examined, of which 5 were eliminated due to a lack of relevant data. Ultimately, 20 studies (24 comparative groups of 25,873 participants) were included (9, 15–33) (**Figure 1**).

Notably, two comparative groups were contained each in two studies (9, 28), and three comparative groups in one study (15). 15 studies were conducted in Asia, specifically in China (n=13) and Thailand (n=2). The remaining 9 were conducted in Europe, specifically in Turkey (n=6), Italy (n=1), Spain (n=1), and Sweden (n=1). They were all published in English in 2017-2025. They covered a wide range of study populations, including adults without specific diseases, children and adolescents, pregnant women, and stroke and tumor patients.

The sample sizes greatly varied, ranging from 86 (19) (Usta et al., Turkey) to 15,746 (18) (Wei et al., China). The participants were aged 13.97-64.73 years averagely. The PLR cutoff was not explicitly reported in all studies except for two: a cutoff of 112.5 (24) (Önen et al.), and 99.72 and 127.92 (28) (Huang et al.). Overall, the included studies had great heterogeneity in the study type, region, sample characteristics, and PLR processing, so the robustness and generalizability of the findings need to be further investigated by subgroup and sensitivity analyses (**Table 1**).

**Table 1:** Basic characteristics.

Author	Study period	Region	Study type	Population	Sample size	Gender		Mean/median age	PLR cutoff	Blood collection standardization	NOS
						Male	Female				
Özyurt et al.	2017-2018	Türkiye	Case control	Adolescents	188	46	142	Depression: 14.47 ± 1.85 Control: 14.46 ± 1.77	NA	Specified	8
Paniagua et al.	NA	Spain	Case control	Adults without specific diseases	292	135	157	Depression: 53.29 ± 10.43 Control: 48.29 ± 11.49	NA	Specified	7
Uçar et al.	Jan. 2017-Mar. 2017	Türkiye	Case control	Adolescents	144	51	93	Depression: 15.64 ± 1.28 Control: 15.24 ± 1.17	NA	Specified	7
Zhou et al. (16)	NA	China	Case control	Adults without specific diseases	912	276	636	Depression: 53.08 ± 9.20 Control: 53.02 ± 6.75	NA	Specified	8
Cai et al.	2014-2016	China	Case control	Adults without specific diseases	209	116	93	Depression: 46.58 ± 13.38 Control: 46.56 ± 12.94	NA	Unspecified	9
Usta et al.	2013-2017	Türkiye	Case control	Adolescents	86	54	32	Depression: 15.21 ± 1.41 Control: 14.82 ± 1.79	NA	Unspecified	7
Bulut et al. (32)	2015-2020	Türkiye	Case control	Adults without specific diseases	188	80	108	Depression: 41.37 ± 13.69 Control: 39.81 ± 12.96	NA	Unspecified	7
Bulut et al. (33)	2015-2020	Türkiye	Case control	Adults without specific diseases	276	107	169	Depression: 46.06 ± 17.39 Control: 44.18 ± 14.83	NA	Specified	7
Önen et al.	2019-2020	Türkiye	Case control	Children and adolescents	148	44	104	Depression: 13.97 ± 1.98 Control: 14.29 ± 1.67	112.5	Unspecified	7
Puangsri et al.	2020-2021	Thailand	Case control	Adolescents	193	61	132	Depression: 20.0 ± 1.1 Control: 20.0 ± 1.3	NA	Unspecified	7
Ninla-aesong et al.	2020-2021	Thailand	Case control	Adults without specific diseases	193	61	132	Depression: 20.44 ± 1.13 Control: 20.42 ± 1.32	NA	Unspecified	8
Zhu et al. (1)	Jan. 2019-Dec. 2019	China	Case control	Adults without specific diseases	297	115	182	Depression: 39.55 ± 11.19 Control: 40.52 ± 8.61	NA	Unspecified	8
Zhu et al. (2)	Jan. 2019-Dec. 2019	China	Case control	Adults without specific diseases	368	142	226	Depression: 40.43 ± 11.04 Control: 40.52 ± 8.61	NA	Unspecified	8
Zhu et al. (3)	Jan. 2019-Dec. 2019	China	Case control	Adults without specific diseases	297	115	182	Depression: 40.34 ± 10.25 Control: 40.52 ± 8.61	NA	Unspecified	8
Wei et al.	2015-2021	China	Case control	Adults without specific diseases	15746	6056	9693	Depression: 44.95 ± 0.193 Control: 42.82 ± 0.157	NA	Specified	8
Liu et al.	Mar. 2023-Oct. 2023	China	Case control	Adults without specific diseases	109	37	72	Depression: 30(Q1 = 23, Q3 = 35) Control: 26(Q1 = 24, Q3 = 30)	NA	Specified	7
Huang et al. (1)	2014-2016	China	Cohort	Ischemic stroke patients	242	157	85	62.6 ± 10.1	99.72	Unspecified	8
Huang et al. (2)	2014-2016	China	Cohort	Ischemic stroke patients	242	136	86	62.6 ± 10.1	127.92	Unspecified	8
Hu et al. (30)	2015-2017	China	Cohort	Ischemic stroke patients	376	224	152	61.37 ± 10.34	NA	Unspecified	7
Zhou et al. (17)	2009-2017	Sweden	Cohort	Perinatal women	4483	0	4483	Depression: 29.2 ± 4.6 Control: 28.5 ± 4.6	NA	Specified	6
Hu et al. (29)	2014-2017	China	Cohort	Ischemic stroke patients	423	272	151	62.58 ± 10.27	NA	Unspecified	8
La Verde et al.	2019-2021	Italy	Cohort	Full-term pregnant women	211	0	211	32	NA	Unspecified	8
Wu et al. (1)	2022-2023	China	Cohort	Tumor patients	250	156	94	Depression: 64.73 ± 6.89 Control: 63.48 ± 7.21	NA	Specified	7
Wu et al. (2)	2022-2023	China	Cohort	Tumor patients	100	57	43	Depression: 64.55 ± 7.35 Control: 63.65 ± 7.14	NA	Specified	7

PLR, platelet-to-lymphocyte ratio; NOS, Newcastle-Ottawa Scale; NA, not available.

### 3.2 Study quality

The NOS score was 6 for one cohort study, and 7–8 for the remaining five cohort studies. All 14 case-control studies had 7–9 points. To sum up, the majority of studies were of high quality (**Supplementary Tables S2**, **S3**).

### 3.3 Meta-analyses

#### 3.3.1 Association of PLR (categorical variable) with incidence of depressive disorders

**Figure 2:**
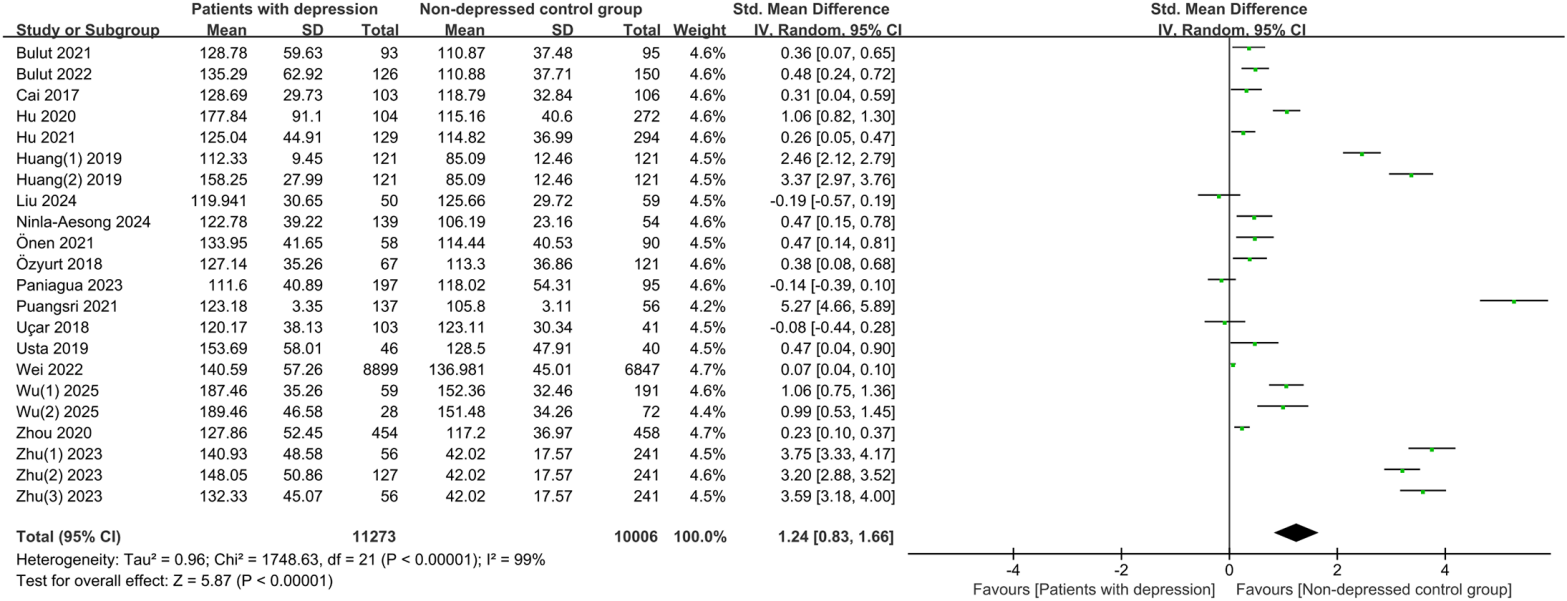
Forest plot for the association of PLR (categorical variable) with incidence of depressive disorders.

The association of PLR as a categorical variable with depressive disorders was examined, involving eight comparative groups of 6,284 participants. A random-effects model was employed. Elevated PLR was associated with an elevated incidence of depressive disorders (OR 1.04, 95% CI 1.00 - 1.08, P = 0.04). CIs of the majority of studies were on the right side of 1, showing statistical significance, and they crossed the null hypothesis in only a few studies. The vast majority of studies reported overall effects in the same direction, and certain heterogeneity was present between studies (I² = 89%) (**Figure 2**).

#### 3.3.2 Association of PLR (continuous variable) with incidence of depressive disorders

**Figure 3:**
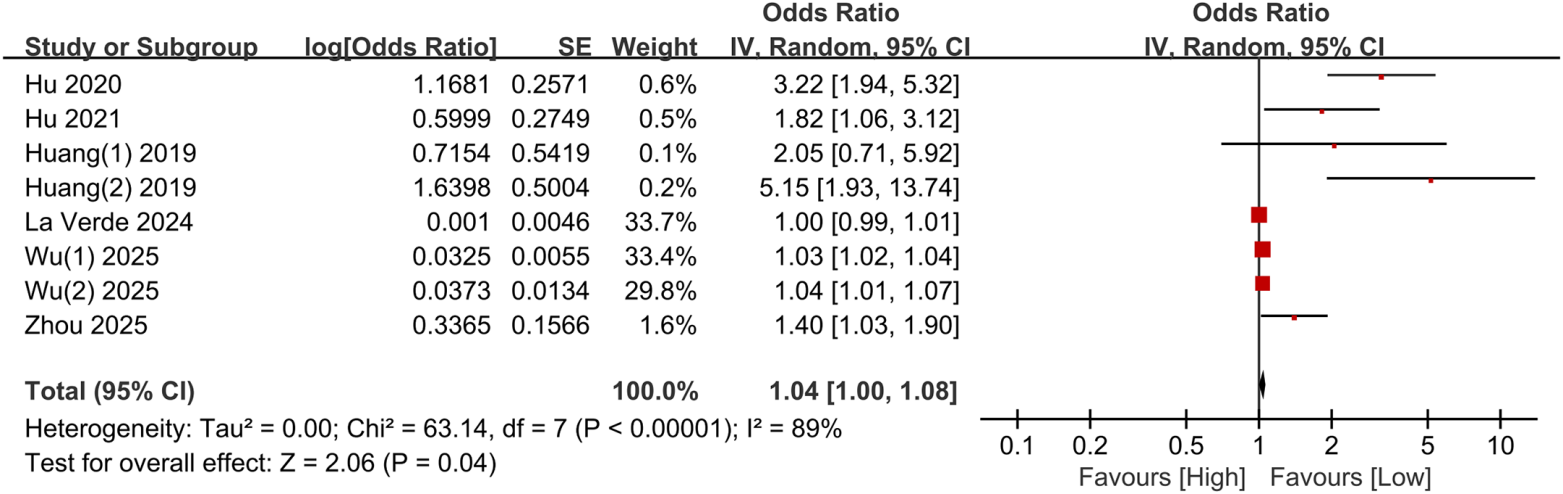
Forest plot for the association of PLR (continuous variable) with incidence of depressive disorders.

Furthermore, the association of PLR as a continuous variable with depressive disorders was investigated, involving 22 studies. PLR was significantly higher in the depressive disorders group (SMD 1.24, 95% CI 0.83 - 1.66, P < 0.00001). CIs of the majority of studies did not cross the null hypothesis, suggesting that PLR was generally higher in depressed patients. CIs crossed the null hypothesis in some studies, but they reported overall effects in the same direction, showing a stable trend of association. Great heterogeneity between studies (I² = 99%, P < 0.00001) indicated large variability in the results (**Figure 3**).

To measure potential heterogeneity, we performed subgroup analyses by sample size, region, study type, and sample characteristics, with SMD and 95% CI calculated. Stratified by study design, the results showed a positive association between PLR levels and the incidence of depressive disorders in both case-control studies and cohort studies (SMD 1.14, 95% CI 0.66–1.61, P = 0.0006; SMD 1.53, 95% CI 0.66–2.39, P < 0.00001), both with high heterogeneity (I² = 98%-99%). Second, the association had a statistically significant difference in both sample size < 200 and ≥ 200 groups (P < 0.05), but both groups had higher heterogeneity (I² ≥ 97%). Third, the association had a statistically significant difference in Asia (SMD 1.36, 95% CI 0.90-1.81, P < 0.00001) and Europe (SMD 0.30, 95% CI 0.10 - 0.49, P = 0.002), and the heterogeneity was higher in Asia (I² = 99%) and lower in Europe (I² = 68%). Notably, the association was the strongest in ischemic stroke patients (SMD 1.78, 95% CI 0.51-3.05, P = 0.006), and the effect size was robust with minimal heterogeneity in tumor patients (SMD 1.04, 95% CI 0.78 - 1.29, P < 0.00001, I² = 0%). In addition, a significant association was also observed in adults without specific diseases (SMD 1.09, 95% CI 0.53 - 1.64, P = 0.0001), but the association did not reach statistical significance in children and adolescents (SMD 1.28, 95% CI - 0.04 to 2.60, P = 0.06). Heterogeneity remained high in most subgroups (I² ≥ 97%), indicating the presence of other undetected influencing factors (**Table 2**).

**Table 2:** Pooled SMDs for PLR in subgroup analyses.

Subgroup	Incidence rate of depressive disorders (Continuous)			
	Study	SMD [95% CI]	P	I^2^
Total	22	1.24 [0.83, 1.66]	<0.00001	99%
Study type				
Cohort	6	1.53 [0.66, 2.39]	<0.00001	98%
Case-control	16	1.14 [0.66, 1.61]	0.0006	99%
Sample size				
<200	9	0.88 [0.18, 1.57]	0.01	97%
≥200	13	1.50 [0.93, 2.07]	<0.00001	99%
Region				
Europe	7	0.30 [0.10, 0.49]	0.002	68%
Asia	15	1.36 [.0.90, 1.81]	<0.00001	99%
Population				
Ischemic stroke patients	4	1.78 [0.51, 3.05]	0.006	99%
Tumor patients	2	1.04 [0.78, 1.29]	<0.00001	0%
Children and adolescents	5	1.28 [-0.04, 2.60]	0.06	98%
Adults without specific diseases	11	1.09 [0.53, 1.64]	0.0001	99%

### 3.4 Sensitivity analysis

**Figure 4:**
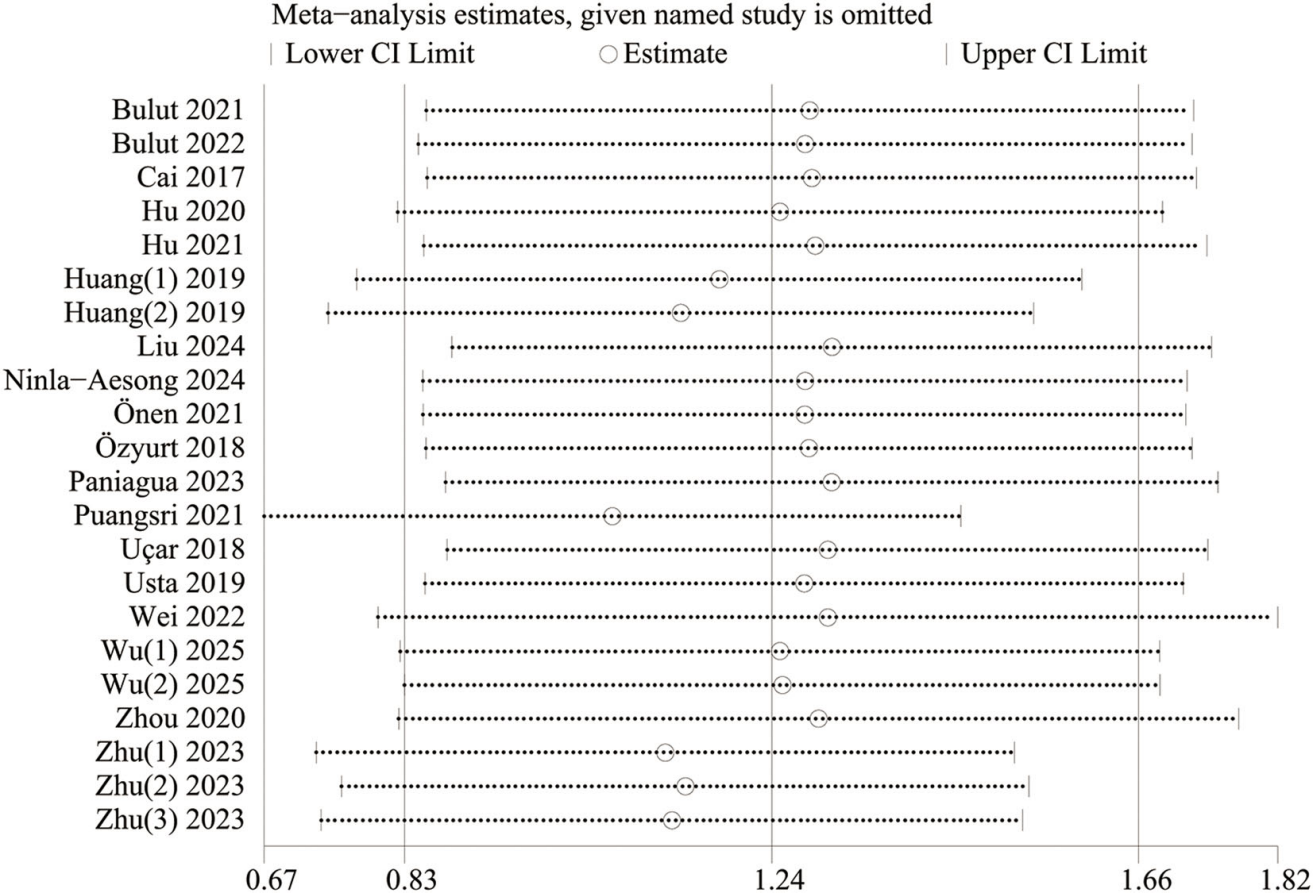
Sensitivity analysis of PLR (continuous variable) and incidence of depressive disorders.

**Figure 5:**
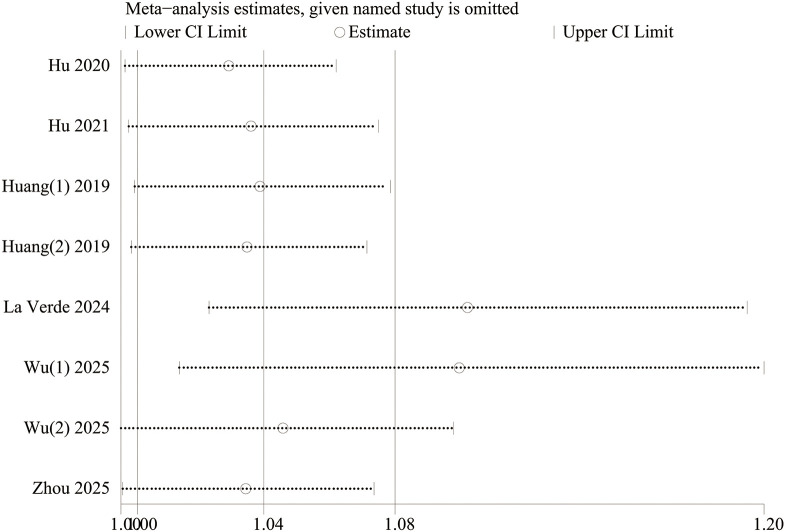
Sensitivity analysis of PLR (categorical variable) and incidence of depressive disorders.

The results were validated for robustness by sensitivity analyses. With PLR as a continuous variable, the effect size estimates fluctuated within 0.67-1.82 after each study was omitted in turn, and they generally remained robust, indicating that no single study had a disproportionate impact on the results, and validating the reliability of the findings (**Figure 4**). With PLR as a categorical variable, the association of PLR with an increased incidence of depressive disorders had statistical significance after four studies (La Verde 2024, Wu(1) 2025, Huang (1) 2019, and Huang (2) 2019) were omitted, but it was not significant after the remaining four studies were omitted (**Figure 5**). To sum up, some heterogeneity was present in the findings, and some studies had a greater impact on the robustness of the pooled results. We can further investigate the sources of heterogeneity to more accurately assess the effect of PLR on the incidence of depressive disorders.

### 3.5 Publication bias

**Figure 6:**
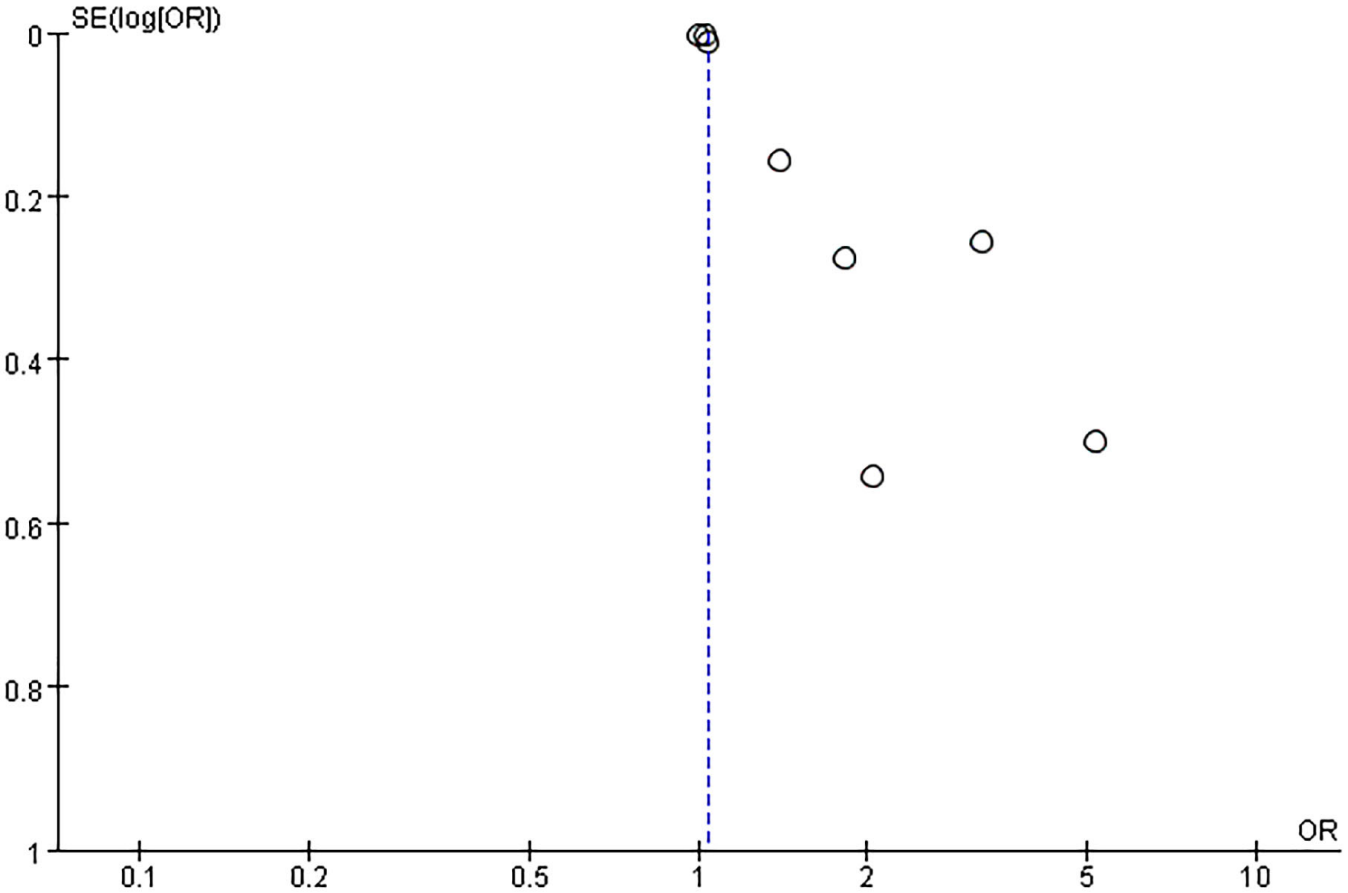
Funnel plot for publication bias for PLR (categorical variable) and incidence of depressive disorders.

**Figure 7:**
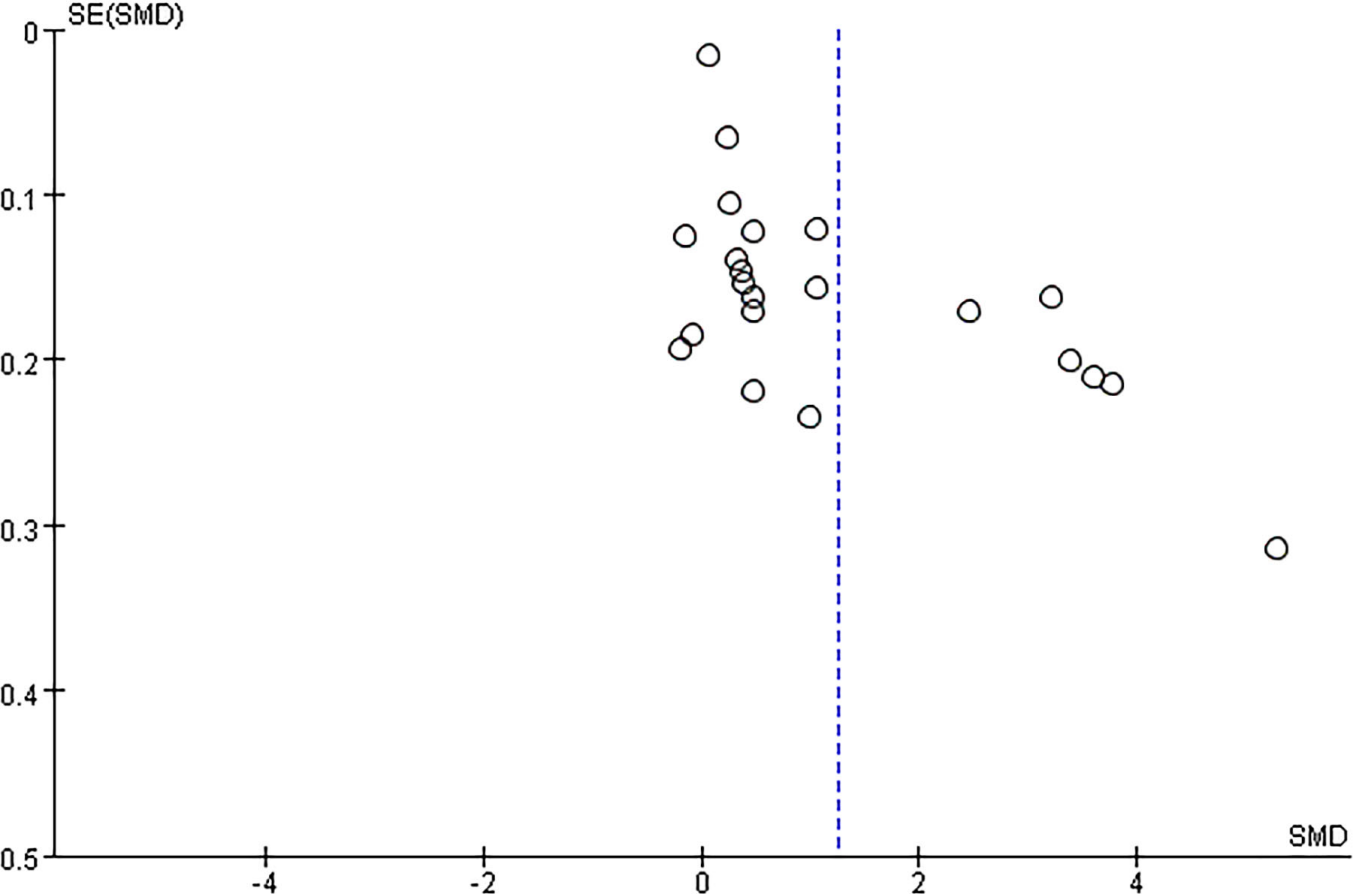
Funnel plot for publication bias for PLR (continuous variable) and incidence of depressive disorders.

**Figure 8:**
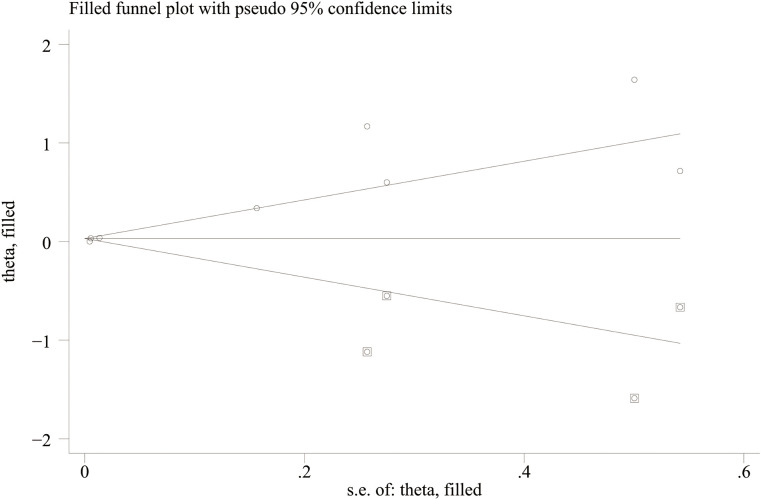
Funnel plot for trim-and-fill method for categorical variables.

**Figure 9:**
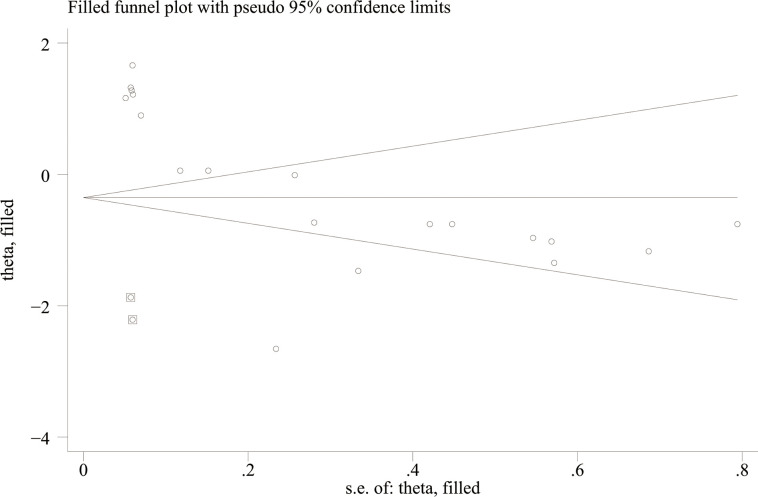
Funnel plot for trim-and-fill method for continuous variables.

Potential publication bias was evaluated using funnel plots and Egger’s test. The results showed possible publication bias when PLR was used as a categorical variable (Egger’s test: P = 0.017) (**Figure 6**) and as a continuous variable (Egger’s test: P = 0.001) (**Figure 7**). The impact of publication bias on categorical and continuous variables was assessed using the trim-and-fill method. After trim-and-fill correction, both categorical variables (OR 1.03, 95% CI 0.98-1.08) (**Figure 8**) and continuous variables (SMD -0.353, 95% CI -1.017 to 0.311) (**Figure 9**) shifted from significant to non-significant. To sum up, selective reporting may be present, especially underreporting of studies with non-significant results.

## 4 Discussion

The involvement of immune and inflammatory pathways in the etiology of depressive disorders has been verified by ample evidence from different research teams (6, 34, 35). Worsened inflammatory responses in the brain are key players in the development of depressive disorders (36). During inflammation in the central nervous system, pro-inflammatory factors and various metabolites also have direct neurotoxicity against the brain (37). These effects may cause disorders of neural circuits related to emotion regulation, cognitive processing, or reward mechanisms, which are closely related to mood and cognitive dysfunction in depressive disorders (38).

This meta-analysis included 24 comparative groups of 25,873 participants to fully investigate the association of PLR with the incidence of depressive disorders, and subgroup and sensitivity analyses were conducted for the robustness of the findings. PLR as a categorical or continuous variable exhibited a positive association with the incidence of depressive disorders, suggesting that PLR can serve as a potential inflammatory marker for identifying high-risk groups of depressive disorders. In addition, with PLR as a continuous variable, the effect size estimates fluctuated less after each study was omitted, indicating that no single study had a decisive influence on the results, and the results were robust. In contrast, with PLR as a categorical variable, some studies had an impact on statistical significance. In the future, further studies are needed to clarify the association of PLR as a categorical variable with the incidence of depressive disorders. Moreover, possible publication bias was observed in the studies when PLR was used as a categorical or continuous variable, possibly attributed to geographic distribution bias or some unpublished negative results, thus overestimating the overall effect. Therefore, the association of PLR with the incidence of depressive disorders remains to be validated by larger-sample clinical studies with more detailed subgroups.

The relationship between inflammation-related indicators and depressive disorders has been previously explored, but few systematic reviews are available on the association of PLR with the incidence of depressive disorders. Cheng et al. (10) included 18 studies in a similar meta-analysis, and found that PLR is significantly higher in depressed patients (n = 2,264) than in controls (n = 2,415), consistent with the findings in this study, but the results were non-robust according to the sensitivity analysis. This study finally included 24 comparative groups of 25,873 participants, improving statistical efficacy and external validity; it optimized the analytical methodology, i.e., dual validation (categorical and continuous variables), compared with previous studies that used only a single effect indicator, so the study framework was more systematic and rigorous. In the future, multiple effects of immune-inflammatory indicators need to be taken into account when establishing depressive disorder prediction models.

Subgroup analyses found a significant positive association of PLR with the incidence of depressive disorders in most participants (e.g., ischemic stroke and tumor patients, and adults without specific diseases), but it did not reach statistical significance in children and adolescents (SMD 1.28, 95% CI - 0.04 to 2.60, P = 0.06), with great heterogeneity (I² = 98%). This suggests that PLR may not be a stable inflammatory predictor in adolescents or that its mechanism differs from that in adults. Adolescence is a phase of neurodevelopment and simultaneous remodeling of the endocrine and immune systems, during which fluctuating hormone levels significantly interfere with the stability of inflammatory indicators. For example, hormonal fluctuations at different adolescent phases significantly influence blood cell ratios, making blood markers of inflammation (including PLR and NLR) ineligible as long-term robust biomarkers (39). The level of peripheral inflammation changes less in adolescents with depressive disorders than in adults, suggesting that the inflammatory response in children and adolescents may be under-activated or still in a compensatory stage (40). Therefore, a single inflammatory indicator such as PLR has limited sensitivity and specificity in children and adolescents. In the future, a multifactorial model should be established, combined with neuroimaging, hormone levels, and social environment, to more accurately assess the inflammatory mechanisms of depressive disorders in adolescents. Multiple studies in adolescent populations suggest that PLR and other peripheral blood inflammatory ratios correlate with the severity of depression and suicidal behavior, indicating that these markers may play roles in disease progression or chronicity. For example, in a study involving 67 depressed adolescents and 121 healthy controls, although overall PLR levels display no significant difference between the two groups, PLR is positively associated with the severity of depression, suggesting that PLR may be involved in the progression or chronicity of depression during adolescence (23). In another study of children and adolescents with depression or anxiety disorders, subjects with a history of suicide attempts have significantly higher baseline PLR levels than those without such a history, suggesting that elevated PLR may serve as a marker for poorer prognosis (41). However, few longitudinal studies have examined the association of PLR changes over time with depressive course (persistence, recurrence, and exacerbation). In the future, multiple PLR measurements are required in prospective designs to investigate whether PLR can predict persistence, recurrence, or exacerbation of depression, and whether PLR decline correlates with clinical improvement. The results of subgroup analysis indicated that heterogeneity significantly decreased among European populations and patients with tumors, suggesting that region and population are the main sources of heterogeneity. Therefore, more international multi-center studies should be conducted and populations should be defined more carefully and specifically in the future to investigate the predictive value of PLR for depressive disorders.

PLR is an economical and easily accessible marker of inflammation, which can be obtained by routine blood tests (27). Wei et al. (18) argued that abnormally activated immune system and chronic inflammation are present in patients with depressive disorders, and inflammatory response can lead to lymphocytopenia and platelet activation, thereby elevating PLR, which may be related to the inflammatory factors affecting platelet and lymphocyte function and count; platelets possess important functions in the inflammatory response, and changes in their count and activity can reflect the inflammatory state. A retrospective cohort study revealed that in depressed patients, the autonomic nervous system and the hypothalamic-pituitary-adrenal axis are activated to promote platelet production, and then activated platelets contribute to thrombosis and endothelial dysfunction, thus participating in the inflammatory response and worsening depression pathology (42). Taniya et al. (43) thought that lymphocytes, the core component of the immune response, may be implicated in the inflammatory cascade by secreting interferon-γ and IL-17. Lymphocytopenia is associated with decreased immunoregulation, which interacts with sustainably activated pro-inflammatory factors to facilitate depressive disorders development and progression (44). In summary, elevated PLR not only reflects the activation status of peripheral inflammation but may also serve as a marker of an imbalance in the inflammatory-immune-neurological pathway, which participates in the depressive disorder progression. In the future, the value of PLR as a potential inflammatory marker in early screening, assessment, and intervention in depressive disorders can be further explored.

However, some limitations are worth noting. First, most of the studies included were retrospective studies, and prospective cohort studies were lacking, which may influence the causal inference. A major limitation of this meta-analysis is the extreme heterogeneity observed across studies (overall I² = 99%), which persisted even after subgroup analyses. Possible sources of heterogeneity included differences in study design (cross-sectional, case-control, or cohort), sample characteristics (age, sex distribution, disease stage and severity), geographic regions, timing and procedures of blood collection, laboratory platforms for PLR measurement, and the cut-off values adopted. In addition, some primary studies provided incomplete reporting, and we were unable to extract corresponding individual-level data, which may have contributed to unexplained variability. Future studies should adopt more unified methodologies and standardized reporting practices, and it is also recommended that they use unified standardized protocols for blood collection and testing, so as to reduce heterogeneity and improve the reliability of conclusions. Additionally, the impact of publication bias was assessed using the trim-and-fill method. The results suggest that publication bias may have influenced the statistical significance of our findings, highlighting the need for more rigorous and unbiased reporting in future studies to obtain more reliable findings. Another limitation of this study is that sex-stratified analyses could not be performed, as most included studies only reported overall PLR values without sex-specific data. Future studies should provide sex-specific results to clarify potential differences. Furthermore, it is recommended that future studies report standardized indicators of severity to facilitate further analysis.

## 5 Conclusion

This meta-analysis indicates that elevated PLR is positively associated with the incidence of depressive disorders. However, given the variability in cut-off values and methodologies among studies, the current evidence is insufficient to recommend PLR as a standalone screening tool. Future studies should aim to standardize PLR cut-off values or report continuous data to enable more nuanced analyses. PLR may reflect peripheral inflammatory status and hold potential value in the early identification or assessment of depressive disorders, but further standardized and prospective studies are warranted. In addition, most included studies were from China, which may restrict the generalizability of our findings, so further studies among diverse populations are needed. Due to limitations and high heterogeneity in the included studies, however, more large-scale prospective cohort studies across races and regions are required in the future to further clarify the potential of PLR as an inflammatory marker in clinical practice.

## Data Availability

The original contributions presented in the study are included in the article/**Supplementary Material**. Further inquiries can be directed to the corresponding author.
