# Supplementary material for: Association of platelet-to-lymphocyte ratio with depression risk: a systematic review and meta-analysis
Source: Front Psychiatry. 2025 Oct 22;16:1671777. doi: 10.3389/fpsyt.2025.1671777 (PMC12586146; doi:10.3389/fpsyt.2025.1671777)
Supplement: Supplementary file 1 [file Table1.docx]

Supplementary Table S1. Literature search strategy

PubMed-141

(((("Depressive Disorder"[Mesh]) OR ((((((((((((((Depressive Disorders) OR (Depressive Neuroses)) OR (Depressive Neurosis)) OR (Endogenous Depression)) OR (Endogenous Depressions)) OR (Melancholia)) OR (Melancholias)) OR (Unipolar Depression)) OR (Unipolar Depressions)) OR (Depressive Syndrome)) OR (Depressive Syndromes)) OR (Neurotic Depression)) OR (Neurotic Depressions)) OR (Depression))) AND (("Blood Platelets"[Mesh]) OR (((((Blood Platelet) OR (Platelets)) OR (Platelet)) OR (Thrombocytes)) OR (Thrombocyte)))) AND (("Lymphocytes"[Mesh]) OR (((Lymphocyte) OR (Lymphoid Cells)) OR (Lymphoid Cell)))) AND (Ratio)

Embase-403

((Depressive Disorder or (Depressive Disorders or Depressive Neuroses or Depressive Neurosis or Endogenous Depression or Endogenous Depressions or Melancholia or Melancholias or Unipolar Depression or Unipolar Depressions or Depressive Syndrome or Depressive Syndromes or Neurotic Depression or Neurotic Depressions or Depression)) and (Blood Platelets or (Blood Platelet or Platelets or Platelet or Thrombocytes or Thrombocyte)) and (Lymphocytes or (Lymphocyte or Lymphoid Cells or Lymphoid Cell)) and Ratio)

Cochrane-13

((Depressive Disorder or (Depressive Disorders or Depressive Neuroses or Depressive Neurosis or Endogenous Depression or Endogenous Depressions or Melancholia or Melancholias or Unipolar Depression or Unipolar Depressions or Depressive Syndrome or Depressive Syndromes or Neurotic Depression or Neurotic Depressions or Depression)) and (Blood Platelets or (Blood Platelet or Platelets or Platelet or Thrombocytes or Thrombocyte)) and (Lymphocytes or (Lymphocyte or Lymphoid Cells or Lymphoid Cell)) and Ratio)

Web of science-161

((((Depressive Disorder) OR ((((((((((((((Depressive Disorders) OR (Depressive Neuroses)) OR (Depressive Neurosis)) OR (Endogenous Depression)) OR (Endogenous Depressions)) OR (Melancholia)) OR (Melancholias)) OR (Unipolar Depression)) OR (Unipolar Depressions)) OR (Depressive Syndrome)) OR (Depressive Syndromes)) OR (Neurotic Depression)) OR (Neurotic Depressions)) OR (Depression))) AND ((Blood Platelets) OR (((((Blood Platelet) OR (Platelets)) OR (Platelet)) OR (Thrombocytes)) OR (Thrombocyte)))) AND ((Lymphocytes) OR (((Lymphocyte) OR (Lymphoid Cells)) OR (Lymphoid Cell)))) AND (Ratio) (Topic)
