# Supplementary material for: Association of platelet-to-lymphocyte ratio with depression risk: a systematic review and meta-analysis
Source: Front Psychiatry. 2025 Oct 22;16:1671777. doi: 10.3389/fpsyt.2025.1671777 (PMC12586146; doi:10.3389/fpsyt.2025.1671777)
Supplement: Supplementary file 2 [file Table2.docx]

Supplementary Table S2 Quality evaluation of the cohort studies with Newcastle–Ottawa scale

| Study | Selection | | | | Comparability | | Outcome | | |
| --- | --- | --- | --- | --- | --- | --- | --- | --- | --- |
|  | Representative-ness | Selection of  non-exposed | Ascertainment  of exposure | Outcome not present at start | Comparability on most important factors | Comparability on other risk factors | Assessment of outcome | Long enough follow-up (median≥1 year) | Adequacy  (completeness) of follow-up |
| Jingjie Hu 2021 | * | * | * | * | * | * | * | - | * |
| Huang Guiqian 2019 | * | * | * | * | * | * | * | - | * |
| Marco La Verde 2024 | * | * | * | * | * | * | * | - | * |
| J. Hu 2020 | * | * | * | * | * | - | * | - | * |
| Xuefeng Wu 2025 | * | * | * | * | * | - | * | - | * |
| J. Zhou 2025 | - | * | * | * | - | - | * | * | * |

*indicates criterion met; - indicates significant of criterion not met
