# Supplementary material for: Association of platelet-to-lymphocyte ratio with depression risk: a systematic review and meta-analysis
Source: Front Psychiatry. 2025 Oct 22;16:1671777. doi: 10.3389/fpsyt.2025.1671777 (PMC12586146; doi:10.3389/fpsyt.2025.1671777)
Supplement: Supplementary file 3 [file Table3.docx]

Supplementary Table S3 Quality evaluation of the case-control with Newcastle–Ottawa scale

| Study | Selection | | | | Comparability | | Measurement of exposure factors | | |
| --- | --- | --- | --- | --- | --- | --- | --- | --- | --- |
|  | Appropriateness of the case | Representative-ness | Comparison selection | Determination of the comparison | Comparability on most important factors | Comparability on other risk factors | Ascertainment  of exposure | Same method of ascertainment | Non-response rate |
| N. S. Bulut 2021 | * | * | * | * | - | - | * | * | * |
| N. S. Bulut 2022 | * | * | * | * | - | - | * | * | * |
| L. Cai 2017 | * | * | * | * | * | * | * | * | * |
| G. Paniagua 2023 | * | * | * | * | - | - | * | * | * |
| G. Özyurt 2018 | * | - | * | * | * | * | * | * | * |
| Y. G. Liu 2024 | * | * | * | * | - | - | * | * | * |
| P. Ninla-Aesong 2024 | * | - | * | * | * | * | * | * | * |
| Ö. Önen 2021 | * | - | * | * | * | - | * | * | * |
| P. Puangsri 2021 | * | - | * | * | * | - | * | * | * |
| H. N. Ucar 2018 | * | - | * | * | * | - | * | * | * |
| M. B. Usta 2019 | * | - | * | * | * | - | * | * | * |
| X. Y. Zhu 2023 | * | * | * | * | * | - | * | * | * |
| Y. Wei 2022 | * | * | * | * | * | - | * | * | * |
| L. Zhou 2020 | * | * | * | * | * | - | * | * | * |

*indicates criterion met; - indicates significant of criterion not met.
